# H5N1 Vaccine-Specific B Cell Responses in Ferrets Primed with Live Attenuated Seasonal Influenza Vaccines

**DOI:** 10.1371/journal.pone.0004436

**Published:** 2009-02-11

**Authors:** Xing Cheng, Michael Eisenbraun, Qi Xu, Helen Zhou, Deepali Kulkarni, Kanta Subbarao, George Kemble, Hong Jin

**Affiliations:** 1 MedImmune, Mountain View, California, United States of America; 2 Laboratory of Infectious Diseases, National Institute of Allergy and Infectious Diseases (NIAID), National Insitutes of Health, Bethesda, Maryland, United States of America; Comprehensive AIDS Reseach Center, China

## Abstract

**Background:**

Live attenuated influenza H5N1 vaccines have been produced and evaluated in mice and ferrets that were never exposed to influenza A virus infection (Suguitan et al., Plos Medicine, e360:1541, 2006). However, the preexisting influenza heterosubtypic immunity on live attenuated H5N1 vaccine induced immune response has not been evaluated.

**Methodology and Principal Findings:**

Primary and recall B cell responses to live attenuated H5N1 vaccine viruses were examined using a sensitive antigen-specific B cell ELISpot assay to investigate the effect of preexisting heterosubtypic influenza immunity on the development of H5N1-specific B cell immune responses in ferrets. Live attenuated H5N1 A/Hong Kong/213/03 and A/Vietnam/1203/04 vaccine viruses induced measurable H5-specific IgM and IgG secreting B cells after intranasal vaccination. However, H5-specific IgG secreting cells were detected significantly earlier and at a greater frequency after H5N1 inoculation in ferrets previously primed with trivalent live attenuated influenza (H1N1, H3N2 and B) vaccine. Priming studies further revealed that the more rapid B cell responses to H5 resulted from cross-reactive B cell immunity to the hemagglutinin H1 protein. Moreover, vaccination with the H1N1 vaccine virus was able to induce protective responses capable of limiting replication of the H5N1 vaccine virus to a level comparable with prior vaccination with the H5N1 vaccine virus without affecting H5N1 vaccine virus induced antibody response.

**Conclusion:**

The findings indicate that previous vaccination with seasonal influenza vaccine may accelerate onset of immunity by an H5N1 *ca* vaccine and the heterosubtypic immunity may be beneficial for pandemic preparedness.

## Introduction

Influenza pandemics can occur when new influenza subtypes capable of both infecting and spreading easily among humans emerge with a new hemagglutinin (HA) subtype (antigenic shift) to which there is little or no population immunity. During the last century, three novel influenza A hemagglutinin subtypes (H1, H2 and H3) have appeared; an H1N1 strain caused the catastrophic “Spanish flu” pandemic in 1918 [1] followed by milder pandemics in 1957 and 1968 caused by H2N2 and H3N2 strains, respectively. Importantly, the origin of the pandemic H2N2 and H3N2 viruses has since been attributed to genetic reassortment events where circulating human influenza viruses acquired novel HA subtypes from avian influenza viruses [2], [3]. Alarmingly, in the past decade, a number of avian influenza viruses containing HA subtypes not typically found in humans have crossed species barrier and infected humans, raising concerns about a future pandemic. Highly pathogenic avian H5N1 influenza viruses have infected only a small number of individuals but are associated with a high mortality rate and are perceived as a potential major global health threat.

Several strategies have been used to develop vaccines against H5N1 viruses including inactivated whole virus vaccines, split or subunit vaccines, live attenuated influenza vaccine (LAIV), vectored vaccines, and DNA vaccines; many of these candidates have shown promise in preclinical studies [4]. Seasonal LAIV has demonstrated several attributes that would be important for an effective pandemic vaccine including efficacy, an ability to protect against antigenically drifted strains, an ability to elicit a rapid immune response in an immunologically naïve population, and a highly efficient production system for the vaccine [5], [6], [7], [8]. Several prototypic pandemic LAIV (pLAIV) 6∶2 reassortant viruses containing the H5N1 HA and NA gene segments have been produced on the backbone of six internal gene segments from the cold-adapted (*ca*) A/Ann Arbor/6/60 vaccine strain [9], the master donor virus (MDV-A) used to produce influenza A vaccine strains for the seasonal FluMist® influenza vaccines (MedImmune). These candidate H5N1 vaccine strains, A/HK/491/97 (HK97 *ca*), A/HK/213/03 (HK03 *ca*), and A/VN/1203/04 (VN04 *ca*), were found to provide complete protection against lethal challenge with homologous and heterologous wild-type (wt) H5N1 viruses in mice and offered complete protection against pulmonary replication of wt H5N1 virus in ferrets [7].

It has been observed that individuals who have recovered from influenza infections develop broad subtype-specific immunity that can protect them from subsequent infection by closely related drift variants of the same subtype [10], [11], [12]. Although not nearly as common, Schulman and Kilbourne [13] reported heterosubtypic immunity in mice, where protection was induced by an influenza virus belonging to a different subtype. Recently, there have been several reports describing heterosubtypic immunity against H5N1 infection induced by influenza virus infection or vaccines in mice [8], [14], [15], [16]. The mechanistic basis of this type of immunity remains undetermined, however, one study demonstrated a role of the N1 component of the vaccine [17] and other studies suggest that structural similarity of the H5 and H1 HA may mediate this type of protection [18], [19]. Kreijtz et al. [20] reported cross-recognition of avian H5N1 influenza virus by human cytotoxic T-lymphocyte populations directed against human influenza viruses and suggested that the preexisting cross-reactive T-cell immunity in humans may dampen the impact of a next pandemic if it is caused by an H5N1 virus. The ferret is considered to be a suitable mammalian host for seasonal influenza vaccine research [21], [22] and for efficacy studies of HPAI H5N1 vaccines [7], [23], [24]. Although ferrets immunized with a H1N1 *ca* strain were not protected from replication of a wild-type H5N1 virus [7], however, because LAIV has been shown to provide protection from strains that are antigenically different from the vaccine antigen, we investigated whether priming with a heterologous seasonal LAIV vaccine containing different subtypes could influence the immune response to H5N1 viruses in the ferret model. Such studies will also help us to understand whether live attenuated H5N1 vaccine could induce effective immune response in individuals that have immunity to seasonal influenza viruses.

HAI and microneutralization assays are frequently used to measure humoral antibody responses, however, these assays may not be sensitive enough to detect early and local antibody responses. To assess the presence and magnitude of heterosubtypic immunity following immunization with LAIV, a sensitive B cell ELISpot assay was developed that could detect early induction of immunity at a time when the HAI assay was less sensitive. Using this assay, we show that local B cell responses induced by the H5N1 VN04 *ca* and HK03 *ca* vaccine viruses can be detected at a virus-specific and HA-specific level. Previous infection with an H1N1 virus induced a faster and higher level B cell response to H5N1 vaccination and could prevent shedding of the H5N1 vaccine virus. The data implies that priming with a non-H5 vaccine may enable a more rapid memory response to an H5 vaccine, however, whether this would be beneficial to the effectiveness of an H5 vaccine remains to be determined.

## Materials and Methods

### Viruses

Influenza virus vaccine strains H1N1 A/New Caledonia/20/99 *ca* (NC99 *ca*), H3N2 A/Wyoming/03/03 *ca* (WY03 *ca*), H3N2 A/California/7/04 *ca* (CA04 *ca*), H5N1 A/Hong Kong/213/03 *ca* (HK03 *ca*), H5N1 A/Vietnam/1203/04 *ca* (VN04 *ca*), H2N2 A/AA/6/60 *ca*, (AA60 *ca* or MDV-A) [22] and H1N2 reassortant *ca* virus containing the H1 HA from A/New Caledonia/20/99 and the N2 NA from A/Wyoming/03/03 were generated by reverse genetics. All viruses were expanded at 33°C for 3 days in the allantoic cavity of 10-day-old embryonated SPAFAS hen's eggs (Norwich, CT). Allantoic fluids collected from infected eggs were examined by hemagglutination assay using 0.5% turkey (tRBC) or horse (hRBC) erythrocytes to determine HA titer. Infectious virus titer was determined by plaque assay (plaque forming unit, PFU) or 50% tissue culture infectious dose (TCID_50_) using Madin-Darby canine kidney (MDCK) cells. Viruses were inactivated by treatment with β-propriolactone (BPL) for use as antigen in ELISpot and ELISA assays. Trivalent LAIV (FluMist®) was manufactured by MedImmune and contained 10^7^ TCID_50_ each of 6∶2 reassortant vaccine strains, A/New Caledonia/20/99 *ca*, A/California/7/04 *ca*, and B/Jilin/20/03 *ca*.

### Animal studies

Ferrets between 7 and 10 weeks of age from Triple F Farms (Sayre, PA) were screened prior to use in experiments for preexisting antibodies to H1N1, H3N2 and H5N1 influenza viruses by hemagglutination inhibition (HAI) assay. Sero-negative ferrets were inoculated intranasally on day 0 with a predetermined does of a monovalent vaccine virus (10^5^ to 10^7^ PFU or TCID_50_ virus), trivalent LAIV, or medium (mock control). To examine the B cell response and virus replication after primary infection, animals were sacrificed 5 or 10 days post-inoculation to collect paratracheal lymph nodes (TLN) and whole blood for B cell ELIspot assays; serum was collected to measure serum antibody level and nasal turbinates were harvested to examine virus replication in the upper respiratory tract. To examine the effect of previous influenza virus infection on the induction of H5N1-specific immune responses, ferrets were given a second intranasal inoculation of 10^7^ or 10^8^ PFU of homologous or heterologous vaccine virus 4–6 weeks after the initial vaccination. Animals in these latter groups were sacrificed 3–10 days later to collect TLN, blood, serum, and nasal turbinate samples or were used to collect serum samples for up to three weeks after the second vaccination. All animal study protocols were approved by MedImmune's Institutional Animal Care and Use Committee and performed in an AAALAC certified facility.

### Measurement of virus titers in animal tissues

Nasal turbinate tissues were homogenized in MEM medium and centrifuged at 400 g for 10 min. Serial 10-fold dilutions of supernatants collected from each preparation were inoculated into three 10- to 11-day old embryonated SPAFAS hen's eggs. After incubation at 33°C for 72 hr, allantoic fluid from each egg was collected for HA assay using 0.5% tRBC. Virus titers in the tissues are reported as a 50% egg infectious dose (EID_50_) per gram of tissue processed.

### B cell ELISpot assay

AcroWell™ 96-well PVDF filter plates (Pall Life Sciences, Ann Arbor, MI) were coated with 50 µL/well PBS containing either 2,000 HA unit/mL of BPL-treated vaccine virus or 10 µg/mL recombinant HA protein derived from H5N1 A/VN/1203/2004 (rH5), H1N1 A/New Caledonia/20/99 (rH1), or H3N2 A/Wyoming/03/03 (rH3) that were purified from recombinant baculovirus infected insect cells (Protein Sciences, Meriden, CT). After overnight incubation at 4°C, plates were washed 3 times with PBS and blocked with RPMI-1640 medium containing 10% FBS for 2 hr at 37°C prior to the addition of cell samples.

Whole blood samples from ferrets were collected in EDTA tubes and processed using Lympholyte®-Mammal (Cedarlane, Ontario, Canada) to isolate peripheral blood mononuclear cells (PBMC). PBMC were washed once with RPMI-1640/10% FBS by centrifugation (300 g for 10 min), counted, and resuspended in complete medium (RPMI-1640, 10% FBS, 2 mM L-glutamine, 0.5 nM ß-mercaptoethanol and penicillin/streptomycin). TLN were harvested from each ferret and placed in cold PBS/5% FBS and the cells were released from TLN into the media by gently rubbing partially minced tissue against a sterile mesh screen with a glass pestle. The resultant cell suspension was collected, passed through a cell strainer to remove large debris, and pelleted by centrifugation (300 g for 10 min). Cell pellets were washed once, counted, and resuspended in the RPMI-1640 complete medium. PBMC and TLN cell suspensions were added to triplicate wells (100 µL/well) at a concentration of 3×10^6^/mL for PBMC or 10^5^ to 10^6^/mL for TLN samples and incubated at 37°C, 5% CO2 for 5 hr. The plates were washed 5 times with PBS containing 0.05% Tween-20 (PBS-T) to remove the cells from the plate. To measure isotype-specific B cell responses, goat anti-ferret IgM (Rockland, Gilbertville, PA) or goat anti-ferret IgG (Bethyl Laboratories, Montgomery, TX) diluted 1∶1000 in PBS-T/1% BSA and incubated overnight at 4°C. After 5 washes with PBS-T, HRP-conjugated rabbit anti-goat Ig (Dako, Carpinteria, CA) diluted 1∶2000 in PBS-T/BSA was added to all wells and incubated at 37°C for 1 hr. Plates were washed 3 times with PBS-T and 3 times with PBS before development with AEC substrate (Vector Labs, Burlingame, CA) for 10 min at room temperature (RT). Wells were rinsed extensively with water and allowed to dry completely before spots in each well were counted using an ImmunoSpot plate reader (Cellular Technologies, Ltd., Cleveland, OH).

### HAI and microneutralization assays

Prior to serologic analysis, ferret sera were treated with receptor-destroying enzyme (RDE) (Denka Seiken, Tokyo, Japan) that was reconstituted with 10 mL of 0.9% NaCl per vial. 0.1 mL serum was mixed with 0.15 mL RDE and incubated at 37°C for 18 hr and adjusted to a final 1∶4 dilution by adding 0.15 mL of 0.9% sodium citrate followed by incubation at 56°C for 45 min. Strain-specific serum HAI titers were determined using 0.5% tRBC or hRBC and the HAI titers are presented as the reciprocal value of the highest serum dilution that did not inhibit hemagglutination. Serum neutralizing antibody titers were determined by microneutralization assay using MDCK cells. RDE-treated ferret serum was 2-fold serially diluted, incubated with 100 TCID_50_ virus at 33°C for 1 hr and transferred onto MDCK cell monolayers in 96-well culture plates (Costar, Corning, NY). After 6 days' incubation at 33°C, the cell monolayers were fixed with 10% formaldehyde, incubated with chicken MDV-A polyclonal antibody followed by incubation with an HRP-conjugated rabbit anti-chicken IgG (Thermo, Rockford, IL), and developed with TMB substrate (Sigma, St. Louis, MO). The reaction was stopped with an equal volume of 0.1 N HCl and the absorbance at 450 nm was determined using a SpectraMax plate reader (Molecular Devices, Sunnyvale, CA). Neutralizing antibody titers were calculated as the highest serum dilution with a value less than that calculated by the formula of (average OD of virus-infected wells - average OD of cell control wells)/2+average OD of cell control wells.

### ELISA analysis of HA-specific antibody responses

96-well EIA plates (Costar, Corning, NY) were coated with 0.025 µg/well of rH1, rH3 or rH5 in PBS overnight at 4°C. Plates were washed 3 times with PBS-T and blocked with SuperBlock Blocking Buffer (Pierce, Rockford, IL) for 1 hr at 37°C. RDE-treated ferret sera were 2-fold serially diluted with PBS-T, transferred to 96-well plates (50 µL/well), and incubated for 1 hr at 37°C. Plates were washed with PBS-T and incubated for 30 min at 37°C with 100 µL/well HRP-conjugated goat anti-ferret IgG (Bethyl Laboratories, Montgomery, TX) diluted 1∶10,000 in PBS-T/1% BSA. After washing with PBS-T, plates were developed with TMB substrate and read as described above in the microneutralization assay. Antibody titers are expressed as the highest dilution with an optical density (OD) reading greater than 2 times the mean OD+standard deviation of similarly diluted negative control samples.

## Results

### Detection of H5N1-specific B cell responses after immunization with pLAIV using a sensitive B cell ELIspot assay

**Figure 1:**
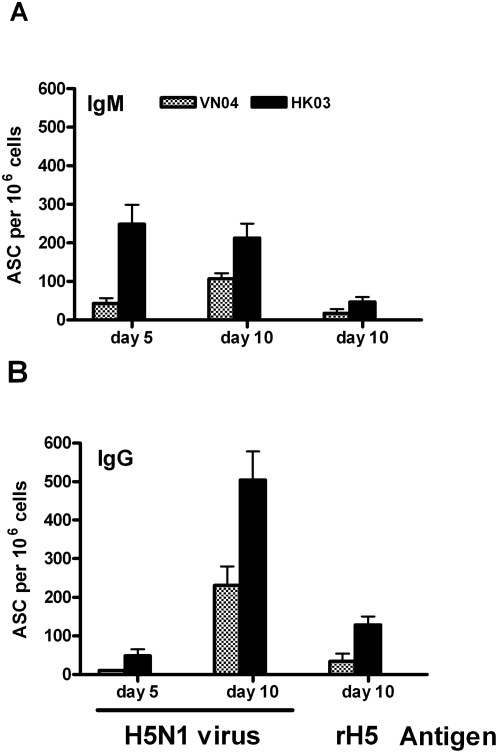
H5N1-specific B cells were detected in ferrets infected with live attenuated H5N1 *ca* vaccines. Ferrets were intranasally administered the H5N1 VN04 *ca* or HK03 *ca* viruses on day 0. Five and ten days post-inoculation, ferrets were sacrificed to collect paratracheal lymph nodes (TLN) and a B cell ELISpot assay was performed using lymphocytes isolated from TLN and BPL-inactivated H5N1 HK03 *ca* virus or rH5 HA antigens. The number of IgM ASC (A) and IgG ASC (B) are presented as per 10^6^ lymphocytes.

Pandemic live attenuated influenza vaccines (pLAIV) developed for H5N1 viruses confer protection against wild-type virus challenge in mice and ferrets [7], however, the level of the serum HAI antibody responses induced by the VN04 vaccine was low. We sought to implement a more sensitive ELISpot assay to measure whole virus- and HA-specific antibody secreting cells (ASC) in draining paratracheal lymph node (TLN) and PBMC from vaccinated ferrets. The ELISpot assay has been shown to be a sensitive tool for detecting cellular immunity following influenza vaccination in humans [25], [26], [27]. To test the sensitivity of this ELISpot assay, ferrets were inoculated intranasally with either the H5N1 VN04 *ca* or H5N1 HK03 *ca* vaccine virus and were sacrificed 5 or 10 days later to isolate TLN cells and PBMC. The ELISpot data obtained from TLN samples (Fig. 1) showed that H5N1 virus-specific ASC could be detected after vaccination with either of the H5N1 *ca* vaccine viruses and similar data were obtained from PBMC (data not shown). However, the magnitude and kinetics of IgM and IgG ASC induced by the VN04 *ca* vaccine appeared to be lower and slower than the HK03 vaccine virus on both days 5 and 10 post-vaccination (Fig 1). The virus-specific IgM ASC response was higher on day 5 than day 10 for HK03 *ca* virus immunized animals, but higher on day 10 than day 5 for VN04 *ca* virus immunized animals, indicating that the initial IgM response induced by the VN04 *ca* virus was slower and weaker than that induced by the HK03 *ca* virus. The IgG secreting ASC response was much higher on day 10 than day 5 for both HK03 *ca* and VN04 *ca* vaccine immunized animals. Again, HK03 *ca* vaccine induced more IgG ASC than VN04 *ca* vaccine (p<0.05). H5 HA-specific IgM and IgG ASC were also detected on day 10 for both viruses. However, the level of HA-specific ASC was only about 15% of the total virus-specific ASC, indicating a majority of the ASC induced by H5N1 *ca* virus vaccine were against antigens other than HA. The level of the H5 HA-specific ASC on day 5 was lower than on day 10 (data not shown). Thus, consistent with the HAI data, the ELISpot data also showed that the HK03 *ca* vaccine induced a better immune response than the VN04 *ca* vaccine.

### LAIV-vaccinated animals show a more rapid immune responses to the H5N1 *ca* vaccine than unvaccinated animals

**Figure 2:**
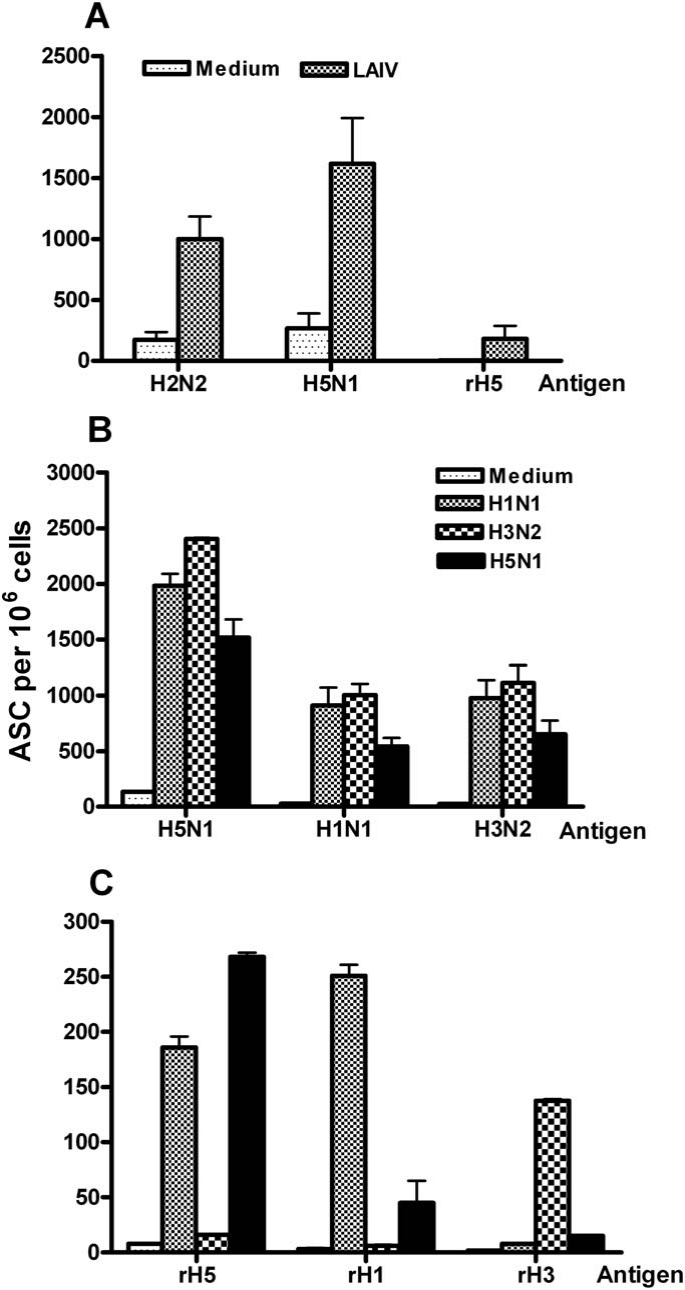
Previous exposure with seasonal LAIV or H1N1 *ca* virus induced a faster H5N1-specific immune response. (A) Ferrets were intranasally inoculated with either medium or trivalent LAIV on day 0. Six weeks later, ferrets were intranasally inoculated with the H5N1 HK03 *ca* vaccine and five days later, lymphocytes isolated from TLN were examined for ASC against H2N2 MDV-A, H5N1 HK03 *ca* viruses and rH5 HA antigens by B cell ELISpot analysis. Ferrets were intranasally inoculated with medium or the monovalent H1N1 NC99, H3N2 CA04, H5N1 HK03 *ca* vaccine viruses on day 0 and intranasally inoculated with the H5N1 HK03 *ca* vaccine six weeks later. The B cell ELISpot analysis was performed with lymphocytes isolated from TLN using the indicated *ca* vaccine virus (B) or rHA (C) as antigens. The IgG antibody secreting B cells are presented as the number of ASC per 10^6^ lymphocytes.

Most people have some immunity to influenza H1N1, H3N2, and B viruses due to previous natural infections or immunization with influenza vaccines. To mimic this sero-positive status and to determine whether preexisting heterosubtypic immunity affects responses to subsequent vaccination with H5N1*ca* vaccine viruses, ferrets were primed intranasally with live attenuated trivalent seasonal influenza vaccines (LAIV) or medium control prior to vaccination with the H5N1 HK03 or VN04 *ca* viruses. Five days after vaccination with H5N1 *ca* vaccine, TLN and PBMC samples were collected for analysis by B cell ELISpot assay to measure virus- and HA-specific IgG ASC. Responses detected using whole virus reagents (Fig. 2A) revealed that the number of H2N2 (AA60, MDV-A) and H5N1 (HK03) virus-specific IgG ASC were significantly higher in ferrets primed with LAIV (Fig. 2A). This was not completely unexpected because both the H1N1 and H3N2 viruses in LAIV share the same internal viral proteins such as the M1 and NP that likely stimulate rapid B cell responses. However, LAIV priming also significantly enhanced H5 HA-specific IgG ASC responses, which were not detected in the unprimed ferrets (Fig. 2A). This finding suggested that the enhanced H5 HA-specific responses could occur as a result of expansion of memory B cells that were elicited against the H1N1 and/or H3N2 influenza A virus components in the LAIV vaccine.

### H1N1 vaccine primes faster B cell responses to the H5N1 vaccine than the H3N2 vaccinated animals

To determine whether the higher numbers of H5-specific B cell responses observed after trivalent LAIV priming were due to only one or both of the influenza A virus components, groups of ferrets were primed with monovalent H1N1 NC99 *ca*, H3N2 CA04 *ca*, H5N1 HK03 *ca* vaccine or medium six weeks prior to a second inoculation with the H5N1 HK03 *ca* vaccine. Five days after vaccination with the H5N1 HK03 *ca* vaccine (Fig. 2B), the level of virus-specific IgG ASC was very low in the ferrets that initially received medium, similar to that observed in the first study. In contrast, ferrets that were primed with the H1N1 NC99, H3N2 CA04 or H5N1 HK03 *ca* vaccine viruses, had significantly higher numbers of H5N1 HK03 *ca* virus-specific IgG ASC after vaccination with the H5N1 HK03 *ca* vaccine. The ferrets that were previously vaccinated with the H1N1, H3N2 and H5N1 *ca* vaccine viruses also had B cell response to the H1N1 and H3N2 vaccine virus (Fig. 2B). The number of the H5 HA-specific IgG ASC was the highest (approximately 10% of total virus-specific ASC) in the group that received 2 doses of HK03 *ca* virus (Fig. 2C). Interestingly, a significant number of H5 HA-specific IgG ASC (approximately 180 ASC per 10^6^ cells) was also observed in the group that previously received the H1N1 NC99 *ca* vaccine virus. A much lower number of ASC was found in the group that received the H3N2 CA04 *ca* vaccine virus. As expected, a significant number of H1 and H3 HA-specific IgG ASC (approximately 250 and 140 per 10^6^ cells, respectively) were detected in the groups that were primed with the H1N1 NC99 and H3N2 CA04 *ca* vaccine viruses, respectively.

### H1N1 *ca* vaccine-induced faster B cell responses to the H5N1 vaccine virus is due to the H1 HA

**Figure 3:**
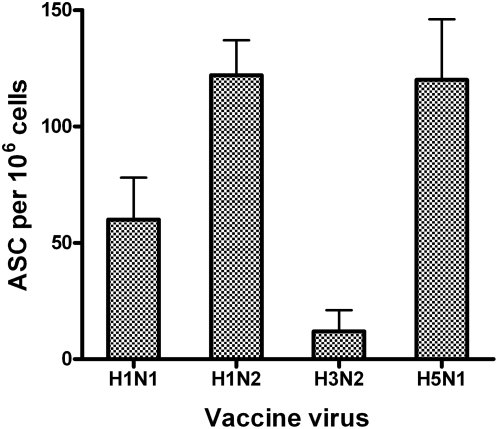
The priming effect of the H1N1 *ca* vaccine is induced by the H1 HA. Ferrets were intranasally inoculated with the H1N1 NC99 *ca* virus, a reassortant H1N2 *ca*, H3N2 CA04 *ca* or H5N1 HK03 *ca* virus on day 0 and intranasally inoculated with the H5N1 HK03 *ca* virus five weeks later. The animals were sacrificed 5 days later and B cell ELISpot analysis was performed with lymphocytes isolated from TLN using rH5 HA as antigen. IgG antibody secreting B cells are presented as the number of ASC per 10^6^ lymphocytes.

The neuraminidases of the H5N1 and H1N1 viruses are of the same (N1) subtype. Several studies have reported a role of the N1 NA-mediated immunity against H5N1 infection in the murine model and in serology analysis of human serum samples [14], [17], [28]. Because it was shown earlier that previous exposure to H3N2 virus did not affect H5-specific ASC responses and the H1 HA and H5 HA share some structural similarity [18] and common epitopes [19], the effect of the H1N1-mediated priming on the H5N1 *ca* vaccines was investigated. To confirm that the enhanced B cell response to the H5N1 virus was due to a primary immune response to the H1 HA not the N1 NA, an H1N2 virus containing the HA from the H1N1 NC99 *ca* virus, the NA from the H3N2 WY03 *ca* virus and the internal protein gene segments from MDV-A was generated. Priming with this H1N2 virus in ferrets elicited a similar level of B cell response (122 ASC per million cells) as those (120 ASC per million cells) primed with the H5N1 HK03 vaccine virus (Fig. 3), confirming that the enhanced H5N1 B cell response was due to the H1 HA and not the N1 NA. The number of the H5 HA-specific IgG ASC in the H1N2 virus primed ferrets was higher than those primed with the H1N1 *ca* vaccine virus, which was possibly due to the better replication of the H1N2 *ca* virus than the H1N1 *ca* virus in the upper respiratory tract of ferrets (data not shown).

### H5-, H1- and H3-specific serum antibody responses following prime-boost vaccination in ferrets

As described earlier, previous exposure to a virus containing the H1 HA resulted in an increased H5-specific B cell response. To examine if the increased ASC response reflected a greater antibody response, serum antibody titers were examined by HAI, microneutralization and ELISA assays. Groups of ferrets were immunized with medium, the H1N1 NC99 *ca*, H3N2 CA04 *ca*, H5N1 HK03 *ca* or VN04 *ca* virus, and 6 weeks later, a second dose of the H5N1 HK03 *ca* or VN04 *ca* vaccine was administered intranasally. Serum samples were collected 6 weeks after the 1^st^ vaccination (post dose 1) and 1 and 3 weeks after the 2^nd^ vaccination (Table 1). At 6 weeks after the first dose neutralizing antibodies against homologous vaccine virus were detected in the ferrets that received vaccine virus but not in the control group that received medium only. The H3N2 CA04 *ca* vaccine induced the highest mean neutralizing antibody (titer of 4064) and the H1N1 NC99 *ca* vaccine induced a mean neutralizing antibody titer of 50. Neither H1N1 nor H3N2 *ca* viruses induced antibodies that cross-neutralized the H5N1 HK03 *ca* virus. The H5N1 HK03 *ca* vaccine induced the H5-specific neutralizing antibody at a mean titer of 320 (Table 1). One week after administration of the H5N1 HK03 *ca* vaccine, the ferrets that received two doses of the HK03 *ca* vaccine had levels of H5-specific neutralizing antibody that were significantly higher than in animals that received a single dose of the H5N1 HK03 *ca* virus. In contrast to the B cell ELISpots results, animals primed with a dose of H1N1 *ca* and boosted with a second dose of H5N1 *ca* for one week had low titers of antibodies against the H5 antigen (Table 1). Previous vaccination with the H3N2 CA04 *ca* vaccine had little effect on the antibody response to the H5N1 *ca* vaccine. When measured at three weeks after vaccination with the H5N1 HK03 *ca* virus, the level of the H5-specific antibody in the animals that received a dose of medium, the H1N1 NC99 or H3N2 CA04 *ca* vaccines as the first dose was much lower (3- to 5-fold) than the animals that received two doses of the H5N1 HK03 *ca* vaccine virus.

**Table 1:** Serum antibody response to influenza viruses after one and two doses of intranasal vaccine.

1^st^ dose vaccine (Day 0)	2^nd^ dose vaccine (Day 42)	Neutralizing antibody GMT against the indicated vaccine virus antigens			
		6 wk post dose-1		1 wk post dose-2	3 wk post dose-2
		1st Vac	H5N1	H5N1	H5N1
Medium	H5N1 HK03 *ca*	<10	<10	40	761
H1N1 NC99 *ca*		50	<10	50	403
H3N2 CA04 *ca*		4064	<10	18	419
H5N1 HK03 *ca*		320	320	2560	2281
Medium	H5N1 VN04 *ca*	<10	<10	14	71
H1N1 NC99 *ca*		63	<10	50	71
H5N1 VN04 *ca*		45	45	806	403

Groups of three ferrets were vaccinated intranasally with the indicated 1^st^ dose of vaccine and 42 days later were inoculated with a 2^nd^ dose of vaccine (H5N1 HK03 *ca* or VN04 *ca*). Serum samples were collected 6 weeks after the 1^st^ dose (pre-dose 2), 1 week and 3 weeks after the 2^nd^ dose, respectively, and antibody titers (geometric mean titers from 3 animals) against the first or second vaccine viruses were determined by microneutralization assay.

The effect of H1N1 *ca* vaccination on the H5N1 VN04 *ca* vaccine induced antibody response was also evaluated (Table 1). As expected, H1N1-specific antibody did not cross-react with the H5N1 VN04 *ca* virus. One week after the second vaccination with the H5N1 VN04 *ca* virus, H5N1-specific neutralizing (titer of 50) antibodies were detected in the ferrets that previously received the H1N1 NC99 *ca* vaccine, and this titer was significantly higher than the ferrets that received medium only (titer of 14, p<0.005) but much lower than the animals that received two doses of the H5N1 VN04 *ca* vaccine (titer of 806). However, three weeks after vaccination with the H5N1 VN04 *ca* vaccine there was no difference in antibody levels between the animals that received the first dose of medium or the H1N1 NC99 *ca* vaccine. Again, animals that received two doses of the H5N1 HK03 *ca* vaccine had neutralizing antibodies more than 5-fold higher than those that received the H1N1 or H3N2 *ca* vaccine as the first dose. Thus, the H1 HA induced enhanced production of H5N1-specific neutralizing antibody was temporary. Similar results were also obtained by the HAI assay (data not shown).

**Figure 4:**
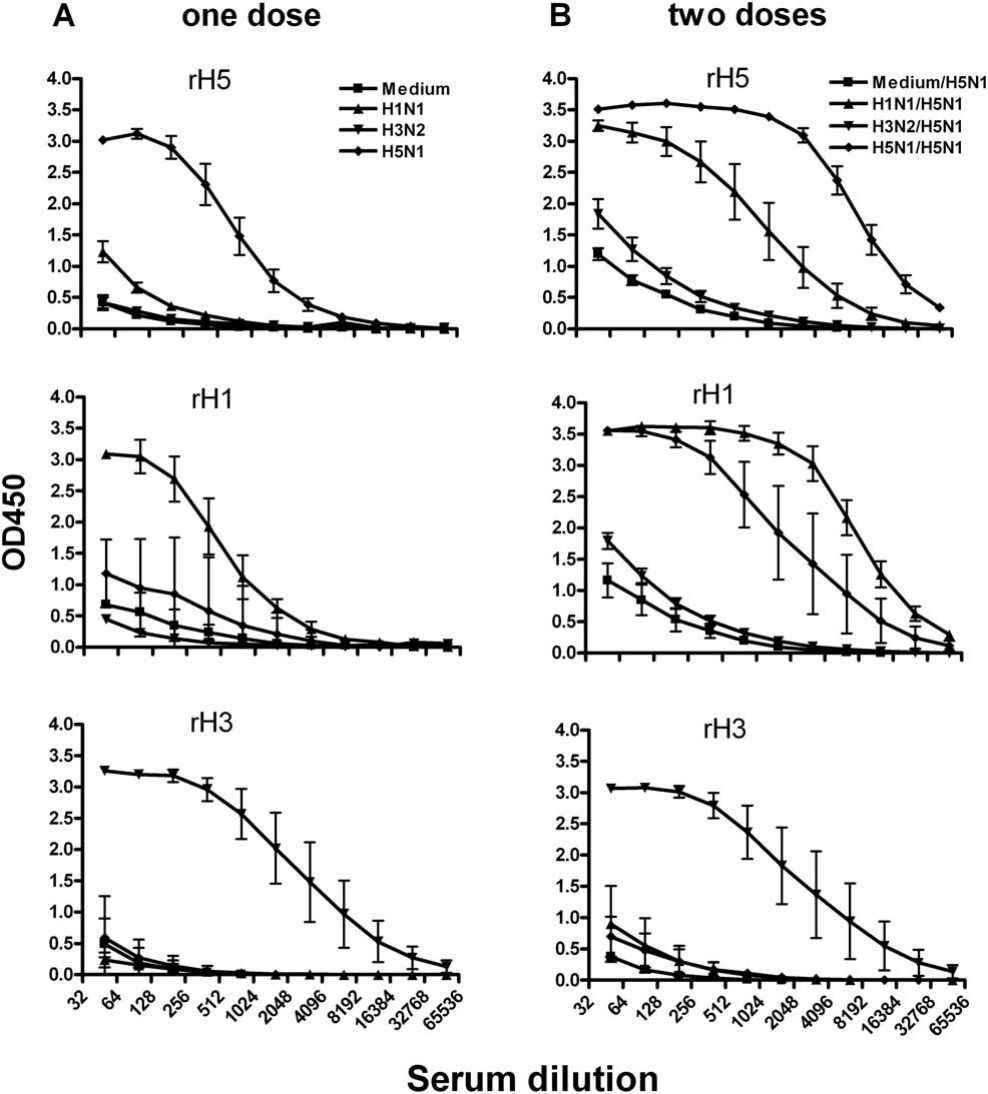
HA-specific antibodies measured by an ELISA assay. Ferrets were intranasally inoculated with medium or the H1N1 NC99, H3N2 CA04, H5N1 HK03 *ca* vaccine viruses on day 0 and intranasally inoculated with the HK03 *ca* vaccine virus six weeks later. Serum samples were collected 6 weeks after the 1st dose (A) and one week after the 2nd dose (B). ELISA was performed with RDE-treated serum using rH5, rH1 or rH3 HA as antigens.

To further evaluate the H1 induced priming effect on the H5N1 antibody response, an ELISA assay was performed to measure levels of HA-specific serum IgG antibodies using rHA as the antigen (Fig. 4). Six weeks after the 1^st^ vaccination, a substantial level of homologous HA-specific IgG was present (Fig. 4A). It was noted that the H1 and H5 specific antibodies had a low level of cross reactivity with the H5 and H1 antigens, respectively. The level of the H5-specific ELISA antibodies was much higher in the group that received the H1N1 NC99 *ca* virus as the first dose and the H5N1 HK03 *ca* virus as the second dose than the ferrets that previously received the H3N2 CA04 *ca* virus and those that did not receive any virus (medium), although the titer was lower than the ferrets that received 2 doses of the H5N1 HK03 *ca* vaccine (Fig. 4B). The antibodies from the ferrets that received 2 doses of the H5N1 HK03 *ca* vaccine also reacted with the rH1 HA. The H5-specific IgG antibodies continued to increase until 3 weeks following the 2^nd^ dose and reached a level that was similar among all the groups (data not shown). Similar data were also obtained for ferrets that received the H5N1 VN04 *ca* vaccine as a second dose (data not shown). Thus, these data indicated that the H1- and H5-specific binding antibodies cross-reacted with each other and previous exposure to H1N1 vaccine appeared to result in a more rapid H5-specific humoral immune response to the H5 HA protein.

### H1N1 vaccine-induced immunity prevented replication of the H5N1 *ca* vaccine virus

To determine whether previous vaccination with the H1N1 NC99 *ca* vaccine would affect subsequent replication of the H5N1 *ca* vaccine virus in the respiratory tract, groups of six naïve ferrets were primed with the H1N1 NC99 *ca*, H3N2 WY03 *ca*, H5N1 HK03 *ca* vaccine or medium only. Four weeks later, animals were inoculated intranasally with the H5N1 HK03 *ca* vaccine and sacrificed on day 3 post-inoculation to collect nasal turbinates to quantify replication of the H5N1 HK03 *ca* vaccine virus in the upper respiratory tract of ferrets. As shown in Table 2, the mean titer of the H5N1 HK03 *ca* vaccine virus detected in the upper respiratory tract of ferrets that had previously received medium was 10^4.5^ EID_50_/g of tissue. In contrast, none of the ferrets that were primed with the H1N1 NC99 *ca* or the H5N1 HK03 *ca* vaccine had detectable virus in the upper respiratory tract. The H3N2 WY03 *ca* vaccine priming protected 2 of 6 ferrets and the H5N1 HK03 virus replicated to a mean titer of 10^2.2^ EID_50_/g of tissue in this group of ferrets. These results indicated that heterosubtypic immunity from H1N1 *ca* virus infection or vaccination reduced H5N1 *ca* vaccine virus replication.

**Table 2:** Effect of the H1N1 and H3N2 *ca* vaccines on replication of the H5N1 HK03 *ca* virus in the upper respiratory tract of ferrets.

Vaccine	# of animals per group	GMT of homologous HAI antibody	# of Animals with H5N1 detected in NT	Mean Virus Titer in NT (log_10_EID_50_/g±SE)
Medium	6	<4	6	4.5±0.5
H1N1 NC99 *ca*	6	47	0	≤1.5
H3N2 WY03 *ca*	6	203	4	2.2±0.7
H5N1 HK03 *ca*	6	40	0	≤1.5

Groups of ferrets were vaccinated with the indicated virus and four weeks later inoculated with the H5N1 HK03 *ca* vaccine. Antibody titers against homologous vaccine virus were determined by HAI assay and expressed as geometric mean titer. The H5N1 HK03 *ca* virus titer in nasal turbinates (NT) on day 3 post-inoculation is expressed as log_10_EID_50_ per gram of tissues calculated from the mean of 6 animals.

## Discussion

In this study, we demonstrate that live attenuated H5N1 vaccine induced immunity can be detected by the B cell ELISpot assay. The protective level of ASC is not well established yet, however, the B cell response to the H5N1 HK03 *ca* vaccine is greater than to the VN04 *ca* vaccine and the magnitude of the ASC response correlates with serum antibody level as determined by microneutralization assay. The H5N1 HK03 and VN04 viruses differ by 9 amino acids in the HA molecule and the amino acid at position 223 is known to contribute to receptor binding specificity [29]. The HA of the HK03 virus that preferentially binds to sialic acid receptors with α2,6-linked oligosaccharide linkages contains serine at residue 223 and the HA of the VN04 virus that prefers an avian-like receptor with α2,3-linked oligosaccharide linkages contains asparagine at this residue [30], [31]. In addition, the length of the NA of the H5N1 HK03 virus differs from the VN04 virus; the VN04 virus, like most of the H5N1 isolates, has a deletion of 20 amino acids in the NA stalk whereas the HK03 virus does not have this deletion. The differences in the HA and NA sequences between the H5N1 HK03 and VN04 viruses presumably contribute to the observed difference in vaccine immunogenicity. Despite the lower immune response induced by VN04 *ca* virus, two doses of the H5N1 VN04 *ca* vaccine offered complete protection against homologous and heterologous H5N1 *wt* virus lethal challenge in mice and provided protection against replication of the H5N1 *wt* virus in the respiratory tracts of mice and ferrets [7].

In addition to the local draining lymph nodes (TLN), ASC were also detected in the PBMC of the vaccinated ferrets at a level slightly lower than those detected in TLN, therefore, only the data obtained with TLN are presented. Virus-specific memory B cells in the lungs can persist for a long time along with germinal center B cells and plasma cells and appear to be a unique feature of the mucosal memory response [16]. In response to re-encountered antigens, memory B cells robustly secrete antibodies against the pathogen and this memory response is much faster than that of primary B cells due to quantitative and qualitative changes in antigen-specific B cells and helper T cells. In this study, we found that previous exposure to the H1N1 *ca* virus could accelerate the memory B cell response to the H5N1 virus. The heterosubtypic antibody response as detected by ELISA in the serum could prevent replication of the H5N1 HK03 *ca* vaccine virus in the respiratory tract, suggesting that protective immunity was enhanced by a priming dose of H1N1 *ca* vaccine. A recent study also showed that the ferrets immunized with the H1N1 virus-like particles (VLP) had a low level of neutralizing antibody against the H5N1 virus and cleared the H5N1 challenge virus rapidly and had reduced morbidity [32]. There was a concern that heterosubtypic immunity might reduce vaccine efficacy by reducing vaccine virus replication in the upper respiratory tract. However, despite restricted replication of the H5N1 *ca* virus in the upper respiratory tract of the H1N1 exposed ferrets, the level of H5N1-specific neutralizing antibodies in the animals that previously received the H1N1 *ca* vaccine was similar to the seronegative animals. Our previous study indicated that two doses of the H1N1 A/New Caledonia/20/99 *ca* virus were unable to protect ferrets from replication of a high dose (10^7^ TCID_50_) of H5N1 HK97 *wt* virus in the respiratory tracts of ferrets [7]. However, it remains to be determined whether the H1N1 *ca* virus could offer a protective benefit from a lower challenge dose of H5N1 *wt* virus. Despite the faster onset of immunity to the H5N1 *ca* vaccine, the antibodies produced in animals that received the H1N1 *ca* vaccine followed by the H5N1 *ca* vaccine are at least 5-fold lower that the animals that received two doses of the H5N1 *ca* vaccines. Thus, it is likely that the immunity provided by previous immunization with an H1N1 *ca* virus is limited and priming with seasonal LAIV cannot replace the use of 2 doses of an H5-specific vaccine.

It has been observed frequently that individuals recovered from influenza virus infection are protected against subsequent infection by antigenic drift variant viruses within the same subtype [10], [11], [12] and to a lesser extent from infection by a different subtype due to heterosubtypic immunity [13]. Recently, several reports have described heterosubtypic immunity from seasonal influenza vaccines to H5N1 infection in mice and humans [8], [14], [15], [16]. Ichinohe et al [14] showed that intranasal inoculation of an inactivated trivalent seasonal influenza vaccine provided cross-protection against H5N1 infection in mice. Such studies are difficult to conduct in humans. Ferrets develop symptoms upon influenza infection that resemble those of humans including sneezing, body temperature variation and weight loss and have been shown to be an appropriate model for influenza virus research. In this study, we demonstrated that the faster H5N1 B cell response induced by the H1N1 *ca* vaccine in ferrets was mediated by the H1 HA protein as demonstrated by a similar effect caused by an H1N2 virus. Although the accelerated H5N1 response following previous exposure to the H1N1 *ca* virus was barely detected by HAI and microneutralization assays, we found a temporal rise of mincroneutralizing antibody in H5N1 VN04 *ca* vaccinated ferrets that were previously exposed to the H1N1 NC04 *ca* virus (Table 1). We could demonstrate cross-reactivity of H1N1 and H5N1 *ca* vaccine induced HA antibodies by ELISA assay. These data confirmed that the H1 and H5 HA contain some conserved epitopes that could elicit cross-reactive antibodies [19], [33] because of their structure similarity [18].

N1 NA-induced protection against experimental H5N1 virus infection has been reported in mice and by the finding that human sera are capable of inhibiting the NA enzymatic activity of the H5N1 VN04 virus [17]. Our study was not designed to examine the contribution of the N1 protein of the H1N1 virus to H5N1 immunity. We cannot exclude the possibility that the N1-induced immunity might also contribute to the restricted replication of the H5N1 *ca* virus in the upper respiratory tract of ferrets.

Our current study indicates that previous exposure to the H3N2 *ca* virus was less protective than the H1N1 *ca* virus in restricting replication of the H5N1 *ca* vaccine virus in the upper respiratory tract of ferrets. However, replication of the H5N1 vaccine virus in ferrets previously primed with an H3N2 *ca* virus was also greatly reduced compared to the control animals. This could be because the H1N1, H3N2 and H5N1 *ca* vaccine viruses share 6 internal protein gene segments. As shown by the ELISpot assay, the number of ASC against the vaccine virus was much higher than HA-specific ASC (compare Fig. 2B with Fig. 2C). In addition to the protective immune response against the HA and NA surface proteins, influenza viruses also induce immune responses against conserved viral proteins such as NP and M1 that could result in heterosubtypic protection [34] to restrict H5N1 virus replication. An earlier report [35] showed that previous mucosal delivery of trivalent influenza vaccine offered protection against H5N1 *wt* virus lethal infection in the mouse model. We also showed previously that H2N2 AA *ca* vaccinated mice were partially protected from the lethal challenge of the H5N1 *wt* viruses [7]. This type of heterosubtypic response could be mediated by secondary CTL responses involving CD8^+^ or CD4^+^ T cells as reported previously [15], [36].

In summary, our study supports the notion that previous vaccination with seasonal influenza vaccine may accelerate onset of immunity by an H5N1 *ca* vaccine. Since the influenza pandemic vaccine may not be available until some time well into the first wave or early in the second wave of a pandemic, an earlier response may be of value in pandemic preparedness.
